# The first high-quality chromosome-level genome of *Parupeneus biaculeatus* using HiFi and Hi-C data

**DOI:** 10.1038/s41597-025-05226-y

**Published:** 2025-06-20

**Authors:** Zhisen Luo, Murong Yi, Xiaodong Yang, Zihao Luo, Xiafang Li, Changping Jiang, Bin Kang, Liangliang Huang, Hung-Du Lin, Xiongbo He, Yunrong Yan

**Affiliations:** 1 College of Fisheries, Guangdong Ocean University, Zhanjiang, 524088 China; 2 Fisheries College, Ocean University of China, Qingdao, 266003 China; 3 College of Environmental Science and Engineering, Guilin University of Technology, Guilin, 541004 China; 4 Guangxi Collaborative Innovation Center for Water Pollution Control and Water Safety in Karst Areas, Guilin, 541004 China; 5 Guangdong Provincial Engineering and Technology Research Center of Far Sea Fisheries Management and Fishing of South China Sea, Guangdong Ocean University, Zhanjiang, 524088 China

**Keywords:** Genome, Ichthyology

## Abstract

*Parupeneus biaculeatus*, also known as pointed goatfish, belongs to the family Mullidae and is distinguished by its unique hyoid barbels containing sensory organs and a specialized foraging strategy, setting it apart from other fish species and making it an ideal model for studying biological adaptations and evolutionary processes. In this study, we present a high-quality chromosome-level genome assembly for *P*. *biaculeatus* using HiFi long reads and Hi-C data. The assembled genome has a total length of 657.58 Mb with a contig N50 of 9.35 Mb, organized into 22 chromosomes covering 99.34% of the genome. A total of 22,490 protein-coding genes were predicted, of which 98.37% were functionally annotated. Repeat analysis revealed that 34.83% of the genome consists of repetitive sequences. The genome assembly achieved an estimated completeness of 99.30% according to BUSCO analysis. This genomic resource provides new opportunities for understanding the biological traits, adaptive mechanisms, and evolutionary history of *P*. *biaculeatus*, and lays a foundation for further genomic studies within the family Mullidae.

## Background & Summary

The family Mullidae comprises 103 species across six genera^1^, widely distributed in the Atlantic, Indian, and Pacific Oceans, with many species being regionally endemic^2–4^. Among them, *Parupeneus biaculeatus*, known as pointed goatfish, is considered endemic to the Western Pacific^5^, primarily distributed from the South China Sea to southern Indonesia. It has been recorded only in China^6^, Japan (northernmost)^7^, Vietnam^8^, and the Lesser Sunda Islands (southernmost)^5^, with particularly abundant populations in southern China, Vietnam, and Japan, making it a key species in these regions. *P*. *biaculeatus* prefers shallow coastal habitats such as rocky reefs, coral reefs, and sandy or muddy substrates, where it exhibits unique foraging adaptations. Like other goatfishes, *P*. *biaculeatus* possesses a pair of independently movable hyoid barbels equipped with chemoreceptors, allowing it to detect benthic invertebrates such as crustaceans, foraminiferans, and polychaetes within the substrate^6^. This specialized foraging behavior is of significant ecological importance, making *P*. *biaculeatus* an ideal subject for studies on foraging behavior and adaptive evolution.

Study on *P*. *biaculeatus* remains very limited despite its distribution along the southern Chinese coast. Current studies have mainly focused on taxonomic morphology^9^ and mitochondrial genome research^10^, with a notable lack of genomic studies. This gap in research has hindered our understanding of the species’ genetic characteristics and adaptive mechanisms. Additionally, the unique barbels of goatfishes and their role in foraging remain poorly understood in terms of adaptive mechanisms^11–13^, and the phylogenetic position of the family Mullidae is still debated. Although traditionally classified within the order Perciformes^14^, recent molecular phylogenetic studies suggest a closer relationship between Mullidae and Syngnathiformes^15–17^. This finding complicates its taxonomic position and highlights the need for more comprehensive genetic research.

To address these issues and lay the foundation for future genetic studies, we have successfully constructed the first chromosome-level reference genome of *P*. *biaculeatus* by integrating high-fidelity (HiFi) long-read sequences and high-throughput chromatin conformation capture (Hi-C) data. The assembled genome has a total length of approximately 657.58 Mb, consisting of 223 contigs with an N50 of 9.35 Mb. The final chromosome-level assembly comprises 22 chromosomes, covering 99.34% of the genome sequence. This high-quality reference genome provides valuable genetic resources for a deeper understanding of the biological characteristics and adaptive mechanisms of *P*. *biaculeatus*, and offers an important reference for further phylogenetic and evolutionary studies within the family Mullidae.

## Methods

### Sample collection and preparation

A female fish (122.38 g) was caught by angling on June 27, 2023, from Kaozhou Bay (22.72°N, 114.96°E), Huidong, Huizhou, Guangdong Province, China. The sampling for this study received approval from the Animal Care and Use Committee of the College of Fisheries at Guangdong Ocean University and was conducted in compliance with their established guidelines and regulations. Dorsal muscle tissues were collected and preserved in liquid nitrogen, from which DNA was subsequently extracted using a modified CTAB method^18^ with RNase A employed to remove RNA contaminants. The quality and quantity of the extracted DNA were examined using a NanoDrop 2000 spectrophotometer (NanoDrop Technologies, Wilmington, DE, USA) and verified via electrophoresis on a 0.8% agarose gel.

### Library construction and sequencing

The SMRTbell library was constructed using the SMRTbell Express Template Prep Kit 2.0. In summary, approximately 15 μg of genomic DNA was mechanically fragmented to an average size of 15 kb using a Covaris g-TUBE, and the fragment size distribution was evaluated with a Femto Pulse system. Large fragment enrichment (>15 kb) was achieved through size selection using a BluePippin device (Sage Science). The quality and concentration of the size-selected libraries were subsequently verified with Femto Pulse and a Qubit Fluorometer (Life Technologies), respectively. Sequencing was conducted on a PacBio Sequel II system utilizing SMRT Cell 8 M (Pacific Biosciences), with each SMRT Cell capturing a 30-hour movie at Frasergen Bioinformatics (Wuhan, China). This process yielded 23.63 Gb of HiFi reads, equating to 35.92 × coverage of the genome (Table 1). Sequencing on a DNBSEQ-T7 platform produced 53.06 Gb of raw data, representing 80.68 × genome coverage (Table 1). Hi-C sequencing was performed to assist in genome assembly and scaffolding, generating 54.10 Gb of data with a coverage depth of 82.26 × (Table 1).

**Table 1:** Sequencing data statistics for *Parupeneus biaculeatus* genome assembly and annotation.

Data type	Tissue	Total base (Gbp)	Reads Numbers	N50 (bp)	Depth (×)
DNBSEQ	Dorsal muscle	53.06	353,733,558	150	80.68
HiFi	Dorsal muscle	23.63	1,424,731	16,775	35.92
Hi-C	Dorsal muscle	54.10	180,340,976	150	82.26

### Genome survey and assembly

**Fig. 1:**
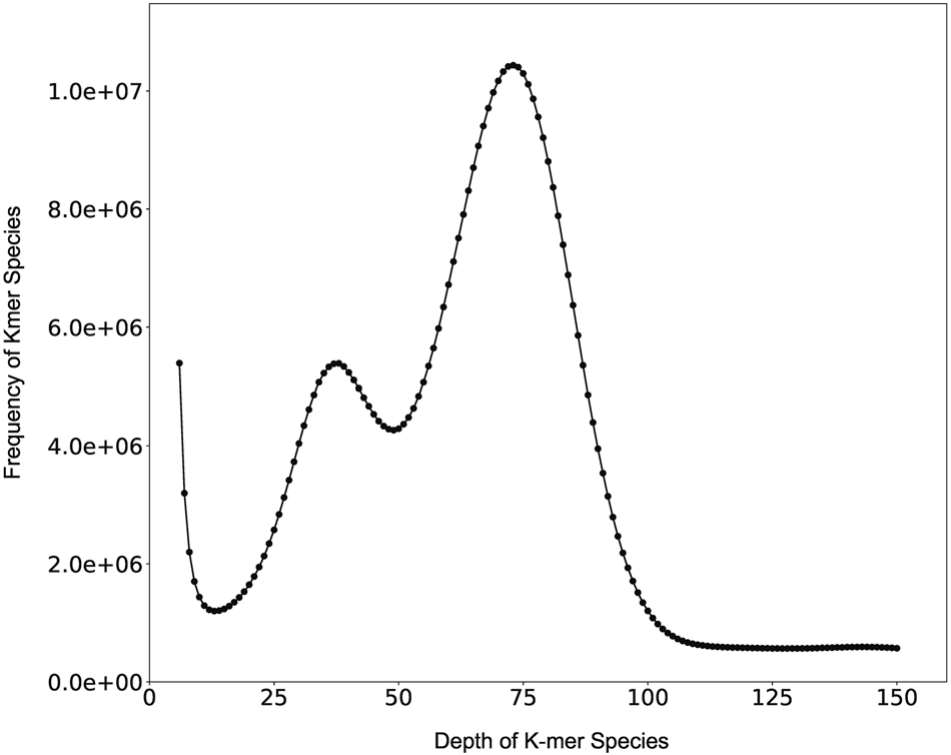
K-mer distribution of *Parupeneus biaculeatus*.

To estimate the genome size, heterozygosity, and repeat content of *P*. *biaculeatus*, we utilized a k-mer frequency approach. Raw reads from the DNBSEQ-T7 platform underwent quality filtering with SOAPnuke (v2.1.0)^19^, using the main parameters (-lowQual = 20, -nRate = 0.005, and -qualRate = 0.5), while other settings remained default. The filtered reads were then analyzed for k-mer frequency (k = 17) using GCE (v1.0.2)^20^ (https://github.com/fanagislab/GCE), estimating the genome size at 586.53 Mb with a peak 17-mer depth of 76. Heterozygosity was estimated at 0.93% (Fig. 1).

For genome assembly, 23.63 Gb of highly accurate (>99%) HiFi reads, corresponding to 35.92 × genome coverage, were assembled using Hifiasm (v0.19.5)^21^ with default parameters. Sequence graphs in GFA format were converted to FASTA using gfatools bubble (v.0.5-r250-dirty, https://github.com/lh3/gfatools). The final assembly of the *P*. *biaculeatus* genome totaled about 657.58 Mb, consisting of 223 contigs with an N50 value of 9.35 Mb.

### Chromosome assignment using Hi-C technology

To construct a chromosome-level genome assembly, Hi-C sequencing data were utilized for scaffold orientation and error correction. High-quality paired-end reads were trimmed with Trimmomatic (v0.40, parameter: LEADING:3 TRAILING:3 SLIDINGWINDOW:4:15 MINLEN:15)^22^ to remove low-quality bases and adapter sequences. The filtered reads were aligned to contigs using Juicer (v3, https://github.com/aidenlab/juicer)^23^ to generate contact frequency matrices. Misjoin correction was performed using 3D-DNA (v180922)^24^ with two iterative rounds (-r2) under default settings. This was followed by manual corrections using Juicebox Assembly Tools (v1.11.08)^23^.

**Fig. 2:**
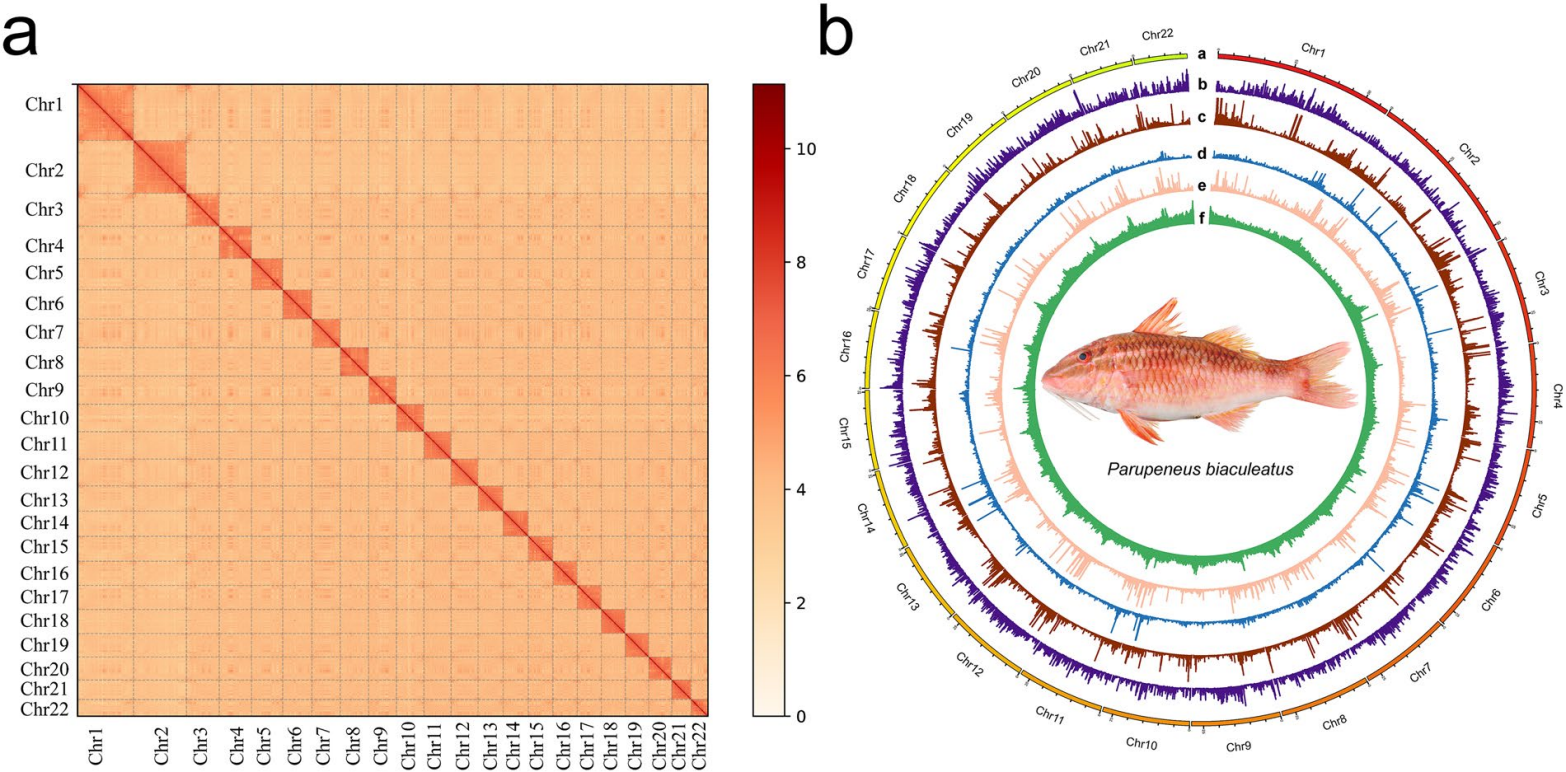
Characterization of assembled *Parupeneus* *biaculeatus* genome. (**b**) Hi-C interaction heatmap for the genome assembly of *P*. *biaculeatus*. Interaction density is displayed by the number of Hi-C read pairs, represented with a color gradient from white (low density) to red (high density). (**b**) Circos plot from outer to inner layers depicts the following: (**a**) Chromosomes (each tick mark represents 5 M); (**b**) gene density; (**c**) LTR density; (**d**) SINE density; (**e**) LINE density; and (**f**) GC density.

This process yielded the first chromosomal-level high-quality assembly, consisting of 22 chromosomes with lengths ranging from 17.20 Mb to 57.98 Mb and accounting for 99.34% of the total sequence (Table 2). The assembly quality was further validated using a heatmap of Hi-C interaction bins (Fig. 2a). The overall genome organization, including gene density and repetitive elements, is illustrated in a Circos plot (Fig. 2b).

**Table 2:** Chromosome size and assembly quality of *Parupeneus biaculeatus* genome evaluated using Merqury for consensus quality value estimation.

Superscaffold	Number of Contigs	Length of Contigs	Length of Superscaffold	QV	Error Rate
Superscaffold1	24	57,975,277	57,986,777	60.6015	9.0e-07
Superscaffold2	10	54,757,966	54,762,466	61.0884	8.0e-07
Superscaffold3	5	34,047,893	34,049,893	59.3054	1.2e-06
Superscaffold4	9	33,579,668	33,583,668	60.7891	8.0e-07
Superscaffold5	6	32,356,289	32,358,789	60.665	9.0e-07
Superscaffold6	8	30,141,245	30,144,745	61.599	7.0e-07
Superscaffold7	4	29,468,478	29,469,978	58.2731	1.5e-06
Superscaffold8	7	29,327,442	29,330,442	60.685	9.0e-07
Superscaffold9	3	28,907,071	28,908,071	59.8457	1.0e-06
Superscaffold10	10	28,394,677	28,399,177	59.4679	1.1e-06
Superscaffold11	5	28,180,074	28,182,074	60.8107	8.0e-07
Superscaffold12	12	27,902,839	27,908,339	59.0513	1.2e-06
Superscaffold13	4	26,053,077	26,054,577	58.9609	1.3e-06
Superscaffold14	5	26,014,186	26,016,186	60.9102	8.0e-07
Superscaffold15	5	25,577,482	25,579,482	60.1658	1.0e-06
Superscaffold16	4	25,164,871	25,166,371	60.5649	9.0e-07
Superscaffold17	8	24,908,726	24,912,226	59.0632	1.2e-06
Superscaffold18	10	24,867,313	24,871,813	58.7336	1.3e-06
Superscaffold19	7	24,380,318	24,383,318	59.2038	1.2e-06
Superscaffold20	8	23,871,359	23,874,859	60.81	8.0e-07
Superscaffold21	5	20,173,717	20,175,717	57.4618	1.8e-06
Superscaffold22	3	17,195,685	17,196,685	59.7105	1.1e-06
TOTAL	162	653,245,653	653,315,653	—	—
ASSEMBLY FINAL	—	—	—	59.6453	1.1e-06

### Repeat annotation

Repetitive sequences, including tandem repeats and transposable elements (TEs), were identified using a combination of de novo and homology-based methods. Tandem repeats were first annotated using Tandem Repeats Finder (TRF, v4.09.1)^25^ with parameters set to 2 7 7 80 10 50 2000. For TEs, identification was performed at both DNA and protein levels. At the DNA level, LTR-RTs were initially identified using LTR_FINDER (v1.0.7, parameters: -w 2 -C)^26^. A de novo repeat library, which included classification information, was generated using RepeatModeler (v2.0.1, default parameters)^27^. RepeatMasker (v4.1.2, parameters: -nolow -no_is -norna -parallel 2)^28^ was then applied to search for TEs by comparing sequences against both the Repbase TE library^29,30^ and the newly constructed de novo library. At the protein level, RepeatProteinMask (v1.36, parameters: -engine ncbi -noLowSimple -pvalue 0.0001) from the RepeatMasker package was used with a WU-BLASTX engine to search the TE protein database. Genome annotation showed that TEs constitute approximately 34.83% of the *P*. *biaculeatus* genome (Table 3).

**Table 3:** Repeat sequence classification statistics of *Parupeneus biaculeatus*.

Type	RepeatMasker TEs Length (bp)	RepeatMasker TEs % in genome	RepeatProteinMask TEs Length (bp)	RepeatProteinMask TEs % in genome	De novo Length (bp)	De novo % in genome	Combined TEs Length (bp)	Combined TEs % in genome
DNA	44073026	6.7	619121	0.09	111041502	16.88	140271987	21.33
LINE	12032828	1.83	3195382	0.49	33728893	5.13	41144996	6.26
SINE	1768872	0.27	0	0	2156456	0.33	3779112	0.57
LTR	12083743	1.84	2521169	0.38	51101634	7.77	60194519	9.15
Other	7811	0	0	0	0	0	7811	0
Unknown	490096	0.07	0	0	31469455	4.79	31928511	4.85
Total TE	62267699	9.47	6333504	0.96	193897984	29.48	229057610	34.83

### Gene structure annotation

We used a combination of homology-based, ab initio, and RNA-seq-assisted annotation to predict the gene structures of *P*. *biaculeatus*.

For homology-based annotation, we aligned coding sequences from related species, including *Synchiropus splendidus*, *Danio rerio*, and *Oryzias latipes*, to the *P*. *biaculeatus* genome using Tblastn (v2.11.0+)^31^. These initial alignments were then refined with Exonerate (v2.4.0, parameters: -model protein2genome)^32^ to enhance the accuracy of the predicted gene models. For RNA-seq-assisted annotation, RNA-seq reads were aligned to the repeat-masked genome using HISAT2 (v2.2.1, default parameters)^33^ to detect splice sites and exonic regions. The resulting alignments were assembled into transcripts with StringTie (v2.1.7, default parameters)^34^. To complement these data, a de novo transcriptome assembly was performed using Trinity (v2.8.5, default parameters)^35^. The PASA pipeline (v2.4.1, default parameters)^36^ was then used to create a comprehensive transcriptome database by integrating all RNA-seq and Iso-seq transcripts. Ab initio gene predictions were generated using multiple tools, including Augustus (v3.4.0, parameters: -noInFrameStop = true -strand = both)^37–39^, Genscan (v1.0)^40^, Geneid (v1.4.4)^41^, and GeneMark (v4.65)^42^, all with default parameters unless specified.

All annotation data were integrated using MAKER (v3.01.03, default parameters)^43^ to generate a comprehensive, nonredundant gene set. The MAKER output was further refined with the PASA pipeline (v2.4.1, default parameters)^36^ to incorporate UTR annotations and identify alternatively spliced isoforms. This thorough approach resulted in a high-quality gene set consisting of 22,490 genes (Table 4).

**Table 4:** Gene annotation statistics of *Parupeneus biaculeatus*.

Gene set	Number	Average gene length (bp)	Average CDS length (bp)	Average exon per gene	Average exon length (bp)	Average intron length (bp)
denovo/AUGUSTUS	21650	14336.09	1629.53	9.23	176.63	1544.79
denovo/Genscan	29385	16035.35	1533.59	8.65	177.39	1896.81
denovo/GeneID	30352	14490.55	1187.68	7.56	157.09	2027.77
denovo/GeneMark	26568	11403.32	1458.44	9.14	159.55	1221.55
homo/*S*. *splendidus*	33932	10193.17	1218.06	6.74	180.75	1563.91
homo/*D*. *rerio*	37810	9793.63	1070.54	5.81	184.38	1814.94
homo/*O*. *latipes*	35813	9449.34	1189.26	6.42	185.38	1525.29
trans.orf/RNAseq	11235	13148.26	1602.42	10.66	272.62	1059.76
MAKER	24021	13865.33	1601.08	9.67	225.06	1348.78
PASA	22490	14860.23	1695.19	10.21	234.8	1343.59

### Gene function annotation

The evaluation of genetic functions is conducted by matching sequences with various databases using Diamond BLASTP (v2.0.7, -evalue 1e-05)^44^. The databases used in this process include the National Center for Biotechnology Information (NCBI) Non-Redundant (NR), Kyoto Encyclopedia of Genes and Genomes (KEGG)^45^, Gene Ontology (GO)^46^, TrEMBL and Swiss-Prot^47^ protein databases. For annotating protein domains, InterProScan (v5.50–84.0, -applications Pfam)^48^ is utilized, based on the InterPro^49^ protein database. Consequently, functional annotations were assigned to 98.37% of the predicted protein-coding genes (Table 5).

**Table 5:** Functional annotation statistics of *Parupeneus biaculeatus*.

Type	Number	Percent(%)
Total	22490	
InterPro	19270	85.68
GO	14827	65.93
KEGG_ALL	21989	97.77
KEGG_KO	15895	70.68
Swissprot	20402	90.72
TrEMBL	21916	97.45
NR	22108	98.3
Annotated	22123	98.37
Unannotated	367	1.63

### Annotation of non-coding RNA genes

We identified transfer RNA (tRNA) genes using the tRNAscan-SE (v2.0.9) algorithm^50^ with default settings. tRNA acts as an adaptor molecule, linking the genetic code in messenger RNA (mRNA) to the amino acids in proteins. For ribosomal RNA (rRNA) sequence prediction, we used RNAmmer (v1.2, parameters: -S euk -multi -m lsu, ssu, tsu)^51^. We also examined small nucleolar RNAs (snoRNAs), which guide the chemical modifications of other RNAs, such as rRNAs, tRNAs, and small nuclear RNAs (snRNAs). MicroRNAs (miRNAs) and snRNAs were detected using Infernal (v1.1.2)^52^, referencing the Rfam database (v14.6)^53^ with default parameters. The analysis revealed 320 miRNAs, 2,311 tRNAs, 1,488 rRNAs, and 500 snRNAs. Altogether, we identified 6,607 non-coding RNAs with an average length of 7,810.83 bp, totaling 894,718 bp, which represents about 0.136% of the genome (Table 6).

**Table 6:** Non-coding RNA annotations statistics of *Parupeneus biaculeatus*.

Type		Copy	Average length(bp)	Total length(bp)	% of genome
miRNA		320	82.92	26533	0.004035
tRNA		2311	75.54	174573	0.026545
rRNA	rRNA	1488	181.89	270656	0.041155
	18S	18	1910.72	34393	0.00523
	28S	15	4608.53	69128	0.010511
	5S	1455	114.87	167135	0.025414
snRNA	snRNA	500	152.3	76150	0.011579
	CD-box	95	113	10735	0.001632
	HACA-box	35	159.86	5595	0.000851
	splicing	366	160.70	58818	0.008944
	scaRNA	4	250.5	1002	0.000152
Total		6607	7810.83	894718	0.136048

### Chromosomal synteny analysis

**Fig. 3:**
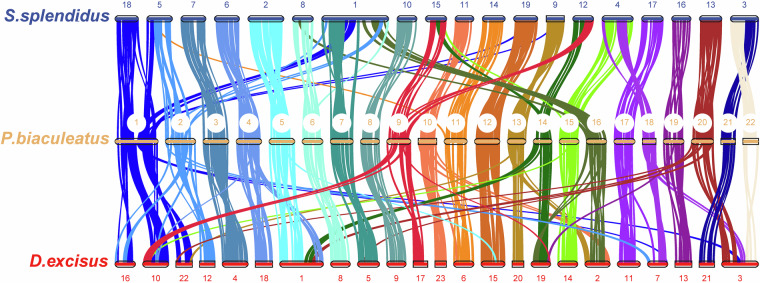
Genomic synteny among *Synchiropus* *splendidus*, *Parupeneus* *biaculeatus* and *Danio* *excisus*.

Molecular phylogenetic studies have confirmed that Mullidae species are closely related to Syngnathiformes species. Therefore, we selected *Doryrhamphus excisus* and *S*. *splendidus* from Syngnathiformes, along with *P*. *biaculeatus*, for chromosomal synteny analysis. The analysis was conducted using TBtools-II (v2.146)^54^, with the One Step MCScanX module was used to identify syntenic relationships, while the Fasta Stats tool generated chromosome backbone data. Synteny visualization was conducted with the multiple synteny plot plugin. The analysis identified several cases of chromosomal fusion and fragmentation among the three species (Fig. 3).

## Data Records

This Whole Genome Shotgun project has been deposited at GenBank^55^ (Accession No.: JBHYRZ000000000.1). The *P*. *biaculeatus* genome project has been submitted to NCBI under BioProject No. PRJNA1154329. The sequencing data from DNBSEQ, PacBio, and Hi-C are available under Accession Nos^56^. SRR30591407, SRR30591406, and SRR30591405, respectively. RNA-seq data can be accessed via Accession No. SRR30591404. The assembled genome data and the genome annotation reported in this paper, with accession number GWHFDQG00000000.1, have been deposited in the Genome Warehouse at the National Genomics Data Center^57,58^ (Beijing Institute of Genomics, Chinese Academy of Sciences / China National Center for Bioinformation) and Figshare^59^, and are publicly accessible at https://ngdc.cncb.ac.cn/gwh and 10.6084/m9.figshare.27080197.

## Technical Validation

To assess the completeness of the assembly and the uniformity of sequencing coverage, we utilized both second-generation (DNBSEQ) and third-generation (HiFi) sequencing data. Using BWA (v0.7.17)^60^ and Minimap2 (v2.14)^61^, we realigned the clean short reads and long reads to the assembled genome. we achieved mapping rates of 99.53% and 99.83%, respectively, and coverage rates (at least 5×) of 99.39% and 99.65% (Table 7). These results highlight the high quality, completeness, and uniformity of the genome assembly.

**Table 7:** Statistics of DNBSEQ and HiFi data remapped to the *Parupeneus biaculeatus* genome.

Data type	Mapping rate (%)	Average sequencing depth	Coverage (%)	Coverage (>=5 × ,%)	Coverage (>=10 × ,%)	Coverage(>=20 × ,%)
DNBSEQ	99.53	77.49	99.80	99.39	98.89	97.52
HiFi	99.83	35.43	99.99	99.65	98.27	88.74

Multiple genome assessments supported the high quality of *P*. *biaculeatus* genome assembly. First, Benchmarking Universal Single-Copy Orthologue (BUSCO, v3.0.2)^62^ analyses showed that 99.30% of the core conserved genes (947 out of 954 metazoa_odb10) were complete in the *P*. *biaculeatus* genome assembly, while 97.7% (932 out of 954) were successfully annotated, indicated the high completeness of the assembled genome (Table 8). Merqury (v1.3) evaluation estimated the consensus quality value (QV) of the assembly at 59.6453 (Table 2).

**Table 8:** Statistics of BUSCO assessment.

Term	Assembly		Annotation	
	Genes	Percentage (%)	Genes	Percentage (%)
Complete BUSCOs	947	99.3	932	97.7
Complete and single-copy BUSCOs	929	97.4	911	95.5
Complete and duplicated BUSCOs	18	1.9	21	2.2
Fragmented BUSCOs	3	0.3	4	0.4
Missing BUSCOs	4	0.4	18	1.9
Total BUSCO groups searched	954	100.0	954	100.0

## Data Availability

No specific code or script was used in this work. Commands used for data processing were all executed according to the manuals and protocols of the corresponding software.

## References

[1] Uiblein, F., Williams, J. T., Bailly, N., Hoang, T. A. & Rajan, P. T. Four new goatfishes (*Upeneus*, Mullidae, Mulliformes) from the Asian Indo-Pacific with a list of valid goatfish species and remarks on goatfish diversity. *Cybium* **48**, 135–160, 10.26028/cybium/2024-001 (2024).

[2] Abliazov, E. R., Boltachev, R., Karpova, ЕP., Pashkov, N. & Danilyuk, N. Ichthyofauna of the Black Sea coastal zone in the Laspi Bay area (Crimea). *Marine Biological Journal* **6**, 3–17, 10.21072/mbj.2021.06.2.01 (2021).

[3] Uiblein, F. & Motomura, H. Three new goatfishes of the genus *Upeneus* from the Eastern Indian Ocean and Western Pacific, with an updated taxonomic account for *U*. *itoui* (Mullidae: *japonicus*-species group). *Zootaxa* **4938**, 298–324, 10.11646/zootaxa.4938.3.2 (2021). 10.11646/zootaxa.4938.3.2 33756973

[4] Echreshavi, S., Esmaeili, H. R. & Jufaili, S. M. A. Goatfishes of the world: An updated list of taxonomy, distribution and conservation status (Teleostei: Mullidae). *FishTaxa* **23**, 1–29 (2022).

[5] Randall, J. E. *Revision of the goatfish genus Parupeneus (Perciformes: Mullidae) with descriptions of two new species*. 1–64 (Bishop Museum, 2004).

[6] Sadovy, Y. & Cornish, A. S. *Reef fishes of Hong Kong*. (Hong Kong University Press, 2000).

[7] Sonoyama, T., Ogimoto, K., Hori, S., Uchida, Y. & Kawano, M. J. B. o. t. K. U. M. An annotated checklist of marine fishes of the Sea of Japan off Yamaguchi Prefecture, Japan, with 74 new records. **11**, 1–152 (2020).

[8] Kimura, S., Imamura, H., Nguyen, V. & Pham, T. *Fishes of Ha Long Bay, the world natural heritage site in northern Vietnam*. ix+314 (Fisheries Research Laboratory, Mie University, 2018).

[9] Tashiro, S. & Motomura, H. First records of *Parupeneus biaculeatus* (Perciformes: Mullidae) from Kagoshima, Japan and comparisons with *Parupeneus ciliatus*. *Nature of Kagoshima* **41**, 133–137, 10.34583/ichthy.5.0_11 (2015).

[10] Luo, Z. *et al*. Mitochondrial genome analysis reveals phylogenetic insights and gene rearrangements in *Parupeneus* (Syngnathiformes: Mullidae). **11**, 1395579, 10.3389/fmars.2024.1395579 (2024).

[11] Hobson, E. S. Feeding relationships of teleostean fishes on coral reefs in Kona, Hawaii. *United States Fishery Bulletin* **72**, 915–1031 (1974).

[12] Gosline, W. A. Structure, function, and ecology in the goatfishes (family Mullidae). *Pac. Sci.* **38**, 312–323 (1984).

[13] Kim, B.-J., Yabe, M. & Nakaya, K. J. I. R. Barbels and related muscles in Mullidae (Perciformes) and Polymixiidae (Polymixiiformes). **48**, 409-413 (2001).

[14] Nelson, J. S., Grande, T. C. & Wilson, M. V. *Fishes of the World*. (John Wiley & Sons, 2016).

[15] Longo, S. J. *et al*. Phylogenomic analysis of a rapid radiation of misfit fishes (Syngnathiformes) using ultraconserved elements. *Mol. Phylogenet. Evol.* **113**, 33–48, 10.1016/j.ympev.2017.05.002 (2017). 28487262 10.1016/j.ympev.2017.05.002

[16] Santaquiteria, A. *et al*. Phylogenomics and historical biogeography of seahorses, dragonets, goatfishes, and allies (Teleostei: Syngnatharia): assessing factors driving uncertainty in biogeographic inferences. *Syst. Biol.* **70**, 1145–1162, 10.1093/sysbio/syab028 (2021). 33892493 10.1093/sysbio/syab028

[17] Nash, C. M., Lungstrom, L. L., Hughes, L. C. & Westneat, M. W. Phylogenomics and body shape morphometrics reveal recent diversification in the goatfishes (Syngnatharia: Mullidae). *Mol. Phylogenet. Evol.* **177**, 107616, 10.1016/j.ympev.2022.107616 (2022). 35998799 10.1016/j.ympev.2022.107616

[18] Gelvin, S. *Plant molecular biology manual*. (Springer Science & Business Media, 2012).

[19] Chen, Y. *et al*. SOAPnuke: a MapReduce acceleration-supported software for integrated quality control and preprocessing of high-throughput sequencing data. *Gigascience* **7**, 1–6, 10.1093/gigascience/gix120 (2018). 29220494 10.1093/gigascience/gix120 PMC5788068

[20] Liu, B., Shi, Y., Yuan, J. & Yuuki, G. Estimation of genomic characteristics by analyzing k-mer frequency in de novo genome projects. *Quantitative Biology* **35**, 62–67, 10.48550/arXiv.1308.2012 (2013).

[21] Cheng, H. *et al*. Haplotype-resolved assembly of diploid genomes without parental data. *Nat. Biotechnol.* **40**, 1332–1335, 10.1038/s41587-022-01261-x (2022). 35332338 10.1038/s41587-022-01261-x PMC9464699

[22] Bolger, A. M., Lohse, M. & Usadel, B. Trimmomatic: a flexible trimmer for Illumina sequence data. *Bioinformatics* **30**, 2114–2120, 10.1093/bioinformatics/btu170 (2014). 24695404 10.1093/bioinformatics/btu170 PMC4103590

[23] Durand, N. C. *et al*. Juicer Provides a One-Click System for Analyzing Loop-Resolution Hi-C Experiments. *Cell Syst* **3**, 95–98, 10.1016/j.cels.2016.07.002 (2016). 27467249 10.1016/j.cels.2016.07.002 PMC5846465

[24] Dudchenko, O. *et al*. De novo assembly of the *Aedes aegypti* genome using Hi-C yields chromosome-length scaffolds. *Science* **356**, 92–95, 10.1126/science.aal3327 (2017). 28336562 10.1126/science.aal3327 PMC5635820

[25] Benson, G. Tandem repeats finder: a program to analyze DNA sequences. *Nucleic Acids Res* **27**, 573–580, 10.1093/nar/27.2.573 (1999). 9862982 10.1093/nar/27.2.573 PMC148217

[26] Xu, Z. & Wang, H. LTR_FINDER: an efficient tool for the prediction of full-length LTR retrotransposons. *Nucleic Acids Res.* **35**, W265–W268, 10.1093/nar/gkm286 (2007). 17485477 10.1093/nar/gkm286 PMC1933203

[27] Flynn, J. M. *et al*. RepeatModeler2 for automated genomic discovery of transposable element families. *Proc. Natl. Acad. Sci. USA* **117**, 9451–9457, 10.1073/pnas.1921046117 (2020). 32300014 10.1073/pnas.1921046117 PMC7196820

[28] Tarailo-Graovac, M. & Chen, N. Using RepeatMasker to identify repetitive elements in genomic sequences. *Curr Protoc Bioinformatics* Chapter 4, Unit 4.10, 10.1002/0471250953.bi0410s25 (2009). 10.1002/0471250953.bi0410s25 19274634

[29] Jurka, J. *et al*. Repbase Update, a database of eukaryotic repetitive elements. *Cytogenet. Genome Res.* **110**, 462–467, 10.1159/000084979 (2005). 16093699 10.1159/000084979

[30] Jurka, J. Repbase update: a database and an electronic journal of repetitive elements. *Trends Genet.* **16**, 418–420, 10.1016/s0168-9525(00)02093-x (2000). 10973072 10.1016/s0168-9525(00)02093-x

[31] De Bie, T., Cristianini, N., Demuth, J. P. & Hahn, M. W. CAFE: a computational tool for the study of gene family evolution. *Bioinformatics* **22**, 1269–1271, 10.1093/bioinformatics/btl097 (2006). 16543274 10.1093/bioinformatics/btl097

[32] Slater, G. S. & Birney, E. Automated generation of heuristics for biological sequence comparison. *BMC Bioinformatics* **6**, 31, 10.1186/1471-2105-6-31 (2005). 15713233 10.1186/1471-2105-6-31 PMC553969

[33] Kim, D., Paggi, J. M., Park, C., Bennett, C. & Salzberg, S. L. Graph-based genome alignment and genotyping with HISAT2 and HISAT-genotype. *Nat. Biotechnol.* **37**, 907–915, 10.1038/s41587-019-0201-4 (2019). 31375807 10.1038/s41587-019-0201-4 PMC7605509

[34] Kovaka, S. *et al*. Transcriptome assembly from long-read RNA-seq alignments with StringTie2. *Genome Biol.* **20**, 278, 10.1186/s13059-019-1910-1 (2019). 31842956 10.1186/s13059-019-1910-1 PMC6912988

[35] Grabherr, M. G. *et al*. Full-length transcriptome assembly from RNA-Seq data without a reference genome. *Nat. Biotechnol.* **29**, 644–652, 10.1038/nbt.1883 (2011). 21572440 10.1038/nbt.1883 PMC3571712

[36] Haas, B. J. *et al*. Improving the Arabidopsis genome annotation using maximal transcript alignment assemblies. *Nucleic Acids Res.* **31**, 5654–5666, 10.1093/nar/gkg770 (2003). 14500829 10.1093/nar/gkg770 PMC206470

[37] Stanke, M., Steinkamp, R., Waack, S. & Morgenstern, B. AUGUSTUS: a web server for gene finding in eukaryotes. *Nucleic Acids Res.* **32**, W309–W312, 10.1093/nar/gkh379 (2004). 15215400 10.1093/nar/gkh379 PMC441517

[38] Stanke, M. & Morgenstern, B. AUGUSTUS: a web server for gene prediction in eukaryotes that allows user-defined constraints. *Nucleic Acids Res.* **33**, W465–W467, 10.1093/nar/gki458 (2005). 15980513 10.1093/nar/gki458 PMC1160219

[39] Stanke, M. *et al*. AUGUSTUS: ab initio prediction of alternative transcripts. *Nucleic Acids Res.* **34**, W435–439, 10.1093/nar/gkl200 (2006). 16845043 10.1093/nar/gkl200 PMC1538822

[40] Burge, C. & Karlin, S. Prediction of complete gene structures in human genomic DNA. *J. Mol. Biol.* **268**, 78–94, 10.1006/jmbi.1997.0951 (1997). 9149143 10.1006/jmbi.1997.0951

[41] Blanco, E., Parra, G. & Guigo, R. Using geneid to identify genes. *Curr Protoc Bioinformatics* Chapter 4, Unit 4 3, 10.1002/0471250953.bi0403s18 (2007). 18428791 10.1002/0471250953.bi0403s18

[42] Borodovsky, M. & McIninch, J. GENMARK: Parallel gene recognition for both DNA strands. *Comput. Chem.* **17**, 123–133, 10.1016/0097-8485(93)85004-v (1993).

[43] Holt, C. & Yandell, M. MAKER2: an annotation pipeline and genome-database management tool for second-generation genome projects. *BMC Bioinformatics* **12**, 491, 10.1186/1471-2105-12-491 (2011). 22192575 10.1186/1471-2105-12-491 PMC3280279

[44] Buchfink, B., Reuter, K. & Drost, H. G. Sensitive protein alignments at tree-of-life scale using DIAMOND. *Nat. Methods* **18**, 366–368, 10.1038/s41592-021-01101-x (2021). 33828273 10.1038/s41592-021-01101-x PMC8026399

[45] Kanehisa, M., Goto, S., Sato, Y., Furumichi, M. & Tanabe, M. KEGG for integration and interpretation of large-scale molecular data sets. *Nucleic Acids Res.* **40**, D109–D114, 10.1093/nar/gkr988 (2012). 22080510 10.1093/nar/gkr988 PMC3245020

[46] Ashburner, M. *et al*. Gene ontology: tool for the unification of biology. The Gene Ontology Consortium. *Nat. Genet.* **25**, 25–29, 10.1038/75556 (2000). 10802651 10.1038/75556 PMC3037419

[47] Boeckmann, B. *et al*. The SWISS-PROT protein knowledgebase and its supplement TrEMBL in 2003. *Nucleic Acids Res.* **31**, 365–370, 10.1093/nar/gkg095 (2003). 12520024 10.1093/nar/gkg095 PMC165542

[48] Jones, P. *et al*. InterProScan 5: genome-scale protein function classification. *Bioinformatics* **30**, 1236–1240, 10.1093/bioinformatics/btu031 (2014). 24451626 10.1093/bioinformatics/btu031 PMC3998142

[49] Mitchell, A. *et al*. The InterPro protein families database: the classification resource after 15 years. *Nucleic Acids Res.* **43**, D213–221, 10.1093/nar/gku1243 (2015). 25428371 10.1093/nar/gku1243 PMC4383996

[50] Lowe, T. M. & Eddy, S. R. tRNAscan-SE: a program for improved detection of transfer RNA genes in genomic sequence. *Nucleic Acids Res.* **25**, 955–964, 10.1093/nar/25.5.955 (1997). 9023104 10.1093/nar/25.5.955 PMC146525

[51] Lagesen, K. *et al*. RNAmmer: consistent and rapid annotation of ribosomal RNA genes. *Nucleic Acids Res.* **35**, 3100–3108, 10.1093/nar/gkm160 (2007). 17452365 10.1093/nar/gkm160 PMC1888812

[52] Nawrocki, E. P., Kolbe, D. L. & Eddy, S. R. Infernal 1.0: inference of RNA alignments. *Bioinformatics* **25**, 1335–1337, 10.1093/bioinformatics/btp157 (2009). 19307242 10.1093/bioinformatics/btp157 PMC2732312

[53] Kalvari, I. *et al*. Rfam 14: expanded coverage of metagenomic, viral and microRNA families. *Nucleic Acids Res.* **49**, D192–D200, 10.1093/nar/gkaa1047 (2021). 33211869 10.1093/nar/gkaa1047 PMC7779021

[54] Chen, C. *et al*. TBtools-II: A “one for all, all for one” bioinformatics platform for biological big-data mining. *Mol. Plant* **16**, 1733–1742, 10.1016/j.molp.2023.09.010 (2023). 37740491 10.1016/j.molp.2023.09.010

[55] Luo, Z., Yi, M., and Yan, Y. *Parupeneus biaculeatus* isolate GOUMuPbia001, whole genome shotgun sequencing project. *GenBank* https://identifiers.org/ncbi/insdc.gca:GCA_042823925.3.

[56] *NCBI Sequence Read Archive* https://identifiers.org/ncbi/insdc.sra:SRP531322

[57] Chen, M. *et al*. Genome Warehouse: A public repository housing genome-scale data. *Genomics Proteomics Bioinformatics* **19**, 584–589, 10.1016/j.gpb.2021.04.001 (2021). 34175476 10.1016/j.gpb.2021.04.001 PMC9039550

[58] Members, C.-N. & Partners Database resources of the National Genomics Data Center, China National Center for Bioinformation in 2024. *Nucleic Acids Res.* **52**, D18–D32, 10.1093/nar/gkad1078 (2024). 38018256 10.1093/nar/gkad1078 PMC10767964

[59] Luo, Z. *Parupeneus biaculeatus* genome annotation. *figshare. Dataset.*10.6084/m9.figshare.27080197 (2025).

[60] Li, H. & Durbin, R. Fast and accurate short read alignment with Burrows-Wheeler transform. *Bioinformatics* **25**, 1754–1760, 10.1093/bioinformatics/btp324 (2009). 19451168 10.1093/bioinformatics/btp324 PMC2705234

[61] Li, H. Minimap2: pairwise alignment for nucleotide sequences. *Bioinformatics* **34**, 3094–3100, 10.1093/bioinformatics/bty191 (2018). 29750242 10.1093/bioinformatics/bty191 PMC6137996

[62] Simao, F. A., Waterhouse, R. M., Ioannidis, P., Kriventseva, E. V. & Zdobnov, E. M. BUSCO: assessing genome assembly and annotation completeness with single-copy orthologs. *Bioinformatics* **31**, 3210–3212, 10.1093/bioinformatics/btv351 (2015). 26059717 10.1093/bioinformatics/btv351

